# Towards an Integrated Framework for Assessing the Vulnerability of Species to Climate Change

**DOI:** 10.1371/journal.pbio.0060325

**Published:** 2008-12-23

**Authors:** Stephen E Williams, Luke P Shoo, Joanne L Isaac, Ary A Hoffmann, Gary Langham

**Affiliations:** University of California, Berkeley, United States of America

## Abstract

Climate change is a major threat to global biodiversity. A novel integrated framework to assess vulnerability and prioritize research and management action aims to improve our ability to respond to this emerging crisis.

Global climate change threatens global biodiversity, ecosystem function, and human well-being, with thousands of publications demonstrating impacts across a wide diversity of taxonomic groups, ecosystems, economics, and social structure. A review by Hughes [1] identified many of the ways that organisms may be affected by and/or respond to climate change. Since then, there has been a dramatic increase in the number of case studies attesting to ecological impacts [2], prompting several recent reviews on the subject (e.g., [3–6]). Several global meta-analyses confirm the pervasiveness of the global climate change “fingerprint” across continents, ecosystems, processes, and species [7–9]. Some studies have predicted increasingly severe future impacts with potentially high extinction rates in natural systems around the world [10,11]. Responding to this threat will require a concerted, multi-disciplinary, multi-scale, multi-taxon research effort that improves our predictive capacity to identify and prioritise vulnerable species in order to inform governments of the seriousness of the threat and to facilitate conservation adaptation and management [12,13].

If we are to minimise global biodiversity loss, we need significant decreases in global emissions to be combined with environmental management that is guided by sensible prioritisation of relative vulnerability. That is, we need to determine which species, habitats, and ecosystems will be most vulnerable, exactly what aspects of their ecological and evolutionary biology determine their vulnerability, and what we can do about managing this vulnerability and minimising the realised impacts. There is an emerging literature on specific traits that promote vulnerability under climate change (e.g., thermal tolerance [14]) as well as a broad literature on the traits that influence species' vulnerability generally (e.g., review by [15]). Less is known about the various mechanisms for either ecological or evolutionary adaptation to climate change, although it is increasingly recognised as a vital component of assessing vulnerability [16,17].

Despite this emerging pool of knowledge, we believe that progress in vulnerability assessment relating to climate change could be hastened if a unified framework was available to coordinate the activities of disparate research disciplines. Specifically, what is needed is a complete working framework for assessing the vulnerability of species that explicitly links: the various components of biotic vulnerability; the regional and local factors determining exposure to climatic change; the potential for both evolutionary and ecological responses, resilience, and active management to mediate the final realised impacts; and the potential for feedback effects. Such a framework would be invaluable as it would integrate and guide thought, research programmes, and policy in the biodiversity/climate change arena and allow significant gaps in knowledge to be clearly identified. To this end, we present a conceptual framework that addresses these challenges (Figure 1).

**Figure 1:**
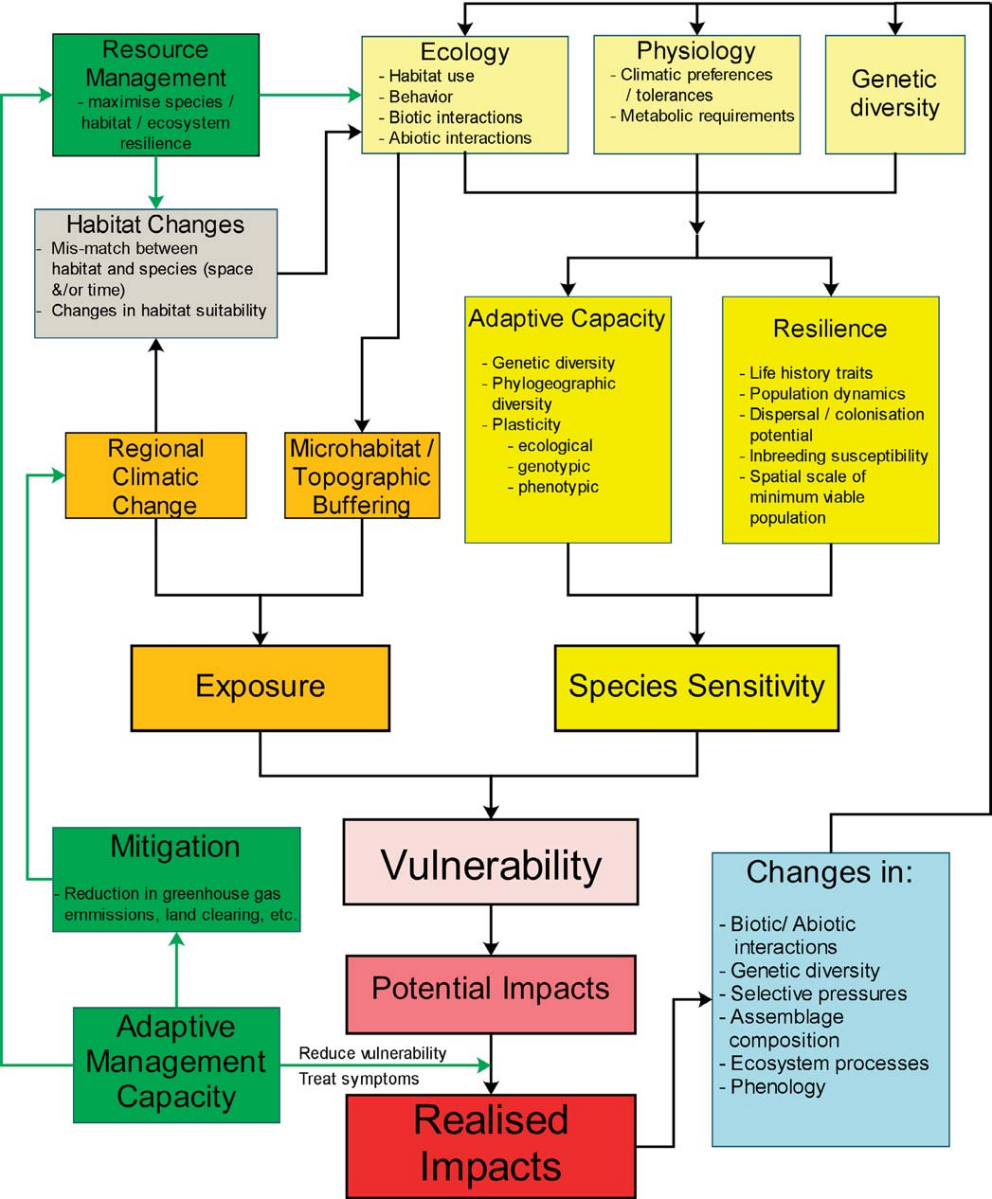
A General Framework To Assess the Vulnerability of Species to Global Climate Change Vulnerability is a function of the species' sensitivity and their exposure to climatic change, mediated by the adaptive potential of the species (both ecological and evolutionary), the resilience of the species, and the capacity for adaptive management to either reduce vulnerability, treat the impacts, mitigate regional exposure, or maximise the system resilience via resource management to increase buffering or reduce other threats. Any realised impacts are likely to cause feedback effects due to changes in biotic/abiotic interaction, loss of genetic diversity, and changes in or loss of ecosystem processes. These feedback effects could result in cascading impacts throughout the ecosystem. All elements of this framework need to be considered in a comprehensive evaluation of vulnerability.

Vulnerability is the susceptibility of a system to a negative impact [18]. A practical first step in assessing vulnerability is to differentiate between factors that determine exposure and factors that govern sensitivity [19,20]. In this context, sensitivity is considered to be governed by traits that are intrinsic to a species and by exposure to factors that are extrinsic to the species and determined by regional climate change and local habitat effects.

## Sensitivity

In the simplest form, sensitivity of a species or individual will be determined by intrinsic factors including physiological tolerance limits, ecological traits (e.g., behaviour), and genetic diversity. However, there is also an increasing recognition that the sensitivity of a species will be mediated by resilience and adaptive capacity (bright yellow panels, Figure 1).

### Ecology, genetic diversity, and physiology.

Clearly some traits that govern sensitivity will be more easily characterised than others. For example, information on relevant ecological traits such as reproductive output are typically available for a broad range of taxa and can be directly incorporated into vulnerability assessments (e.g., [15]). In contrast, for most organisms there is scant information on physiological tolerances and genetic diversity. For these traits, the best interim solution will be to make informed estimates based on the best available ecological knowledge of closely related species on how relevant traits, such as thermal tolerance, may vary across species and over large geographical, temporal, and phylogenetic scales (e.g., [21]).

For example, thermal tolerances of many organisms have been shown to be proportional to the magnitude of temperature variation they experience: lower thermal limits differ more among species than upper thermal limits [22], and upper thermal tolerance is often positively related to acclimatory ability ([23], but see [24]). Importantly, physiological limits are often phylogenetically constrained [25,26], meaning tolerances may only need to be estimated for representatives of many taxa. Similar guiding principles could potentially be developed for genetic diversity. That is, species with restricted ranges or small populations are predicted to have reduced capacity to adapt to environmental change [27], both because genetic variation and potential response to selection should be positively correlated with population size and because individuals should have lower fitness, owing to genetic problems such as inbreeding [28,29]. However, at present, empirical support for these predictions is mixed and different rules may be required for particular scenarios such as naturally small populations that have become inbred over extended periods of time (e.g., small isolated islands), because of potential for partial purging of genetic load and prior elimination of inbreeding depression [30].

Two additional factors will determine the extent to which sensitivity of individuals within a species will affect the vulnerability of the species as a whole. These are the degree of resilience of the species and the capacity of the species to adapt via either ecological or evolutionary responses, potentially modifying their physiological tolerance limits (Figure 1).

### Resilience.

Resilience is the ability of a species to survive and recover from a perturbation. The life history traits that are predicted to promote resilience and recovery and reduce extinction risk include high reproductive rates, fast life history, and short life span [15]. Generally, species with large range sizes tend also to be resistant to extinction, but this may not necessarily be the case in the context of climate change. Large spatial requirements (i.e., spatial scale of operation) may actually be disadvantageous to species in so far as they reduce the likelihood that species will be able to maintain a viable population size in small refugia that are buffered from climatic change, for example in cooler gorges or boulder field habitats. Finally, the ability to disperse within and across habitats, to track the preferred climate space, and to expand rapidly following disturbance will depend on both reproductive rates and dispersal ability [31].

### Adaptive capacity.

Adaptive capacity in its broadest sense includes both evolutionary changes and plastic ecological responses; in the climate change literature, it also refers to the capacity of humans to manage, adapt, and minimise impacts. All organisms are expected to have some intrinsic capacity to adapt to changing conditions; this may be via ecological (i.e., physiological and/or behavioural plasticity) or evolutionary adaptation (i.e., through natural selection acting on quantitative traits). There is now evidence in the scientific literature that evolutionary adaptation has occurred in a variety of species in response to climate change over relatively short time spans (e.g., five to 30 years [16]). However, this is unlikely to be the case for the majority of species and, additionally, the capacity for evolutionary adaptation is probably the most difficult trait to quantify across many species. There is little guidance available at present in terms of making indirect estimates of adaptive capacity.

Evolutionary models have been developed to predict conditions when adaptation to rapid environmental change might be restricted (e.g., [32]). Models predict that the ability to adapt to changing environmental variables depends on a balance between sufficient heritable variation, indirect effects of population size on evolution, and the rate of environmental change [33]. Specialist species are likely to be particularly vulnerable since heritable variation for traits that limit distributions can be low, limiting evolutionary potential under changing environmental conditions even when population sizes are large and there is abundant variation at neutral loci [34]. Even where genetic variation exists, evolutionary changes can be limited by interactions among traits that limit the direction of evolution [28,35,36]. In addition, environmental conditions influence the expression of heritable variation and thereby limit evolutionary responses [37].

An obvious challenge is to identify ecological correlates of evolutionary potential and to undertake long-term studies that can separate genetic from plastic components of adaptive responses. In most cases, ecological plasticity is likely to be more important than evolutionary potential in regard to minimisation of impacts in the short term. This is because plasticity acts within a generation, whereas evolutionary genetic changes involve multiple generations. Studies have demonstrated a number of ways that species have already used pre-existing flexibility to respond to a changing climate, including shifts in distribution, contraction to refugia, shifts in temporal activity (diel and seasonal), acclimation, shifts in habitat/microhabitat use, and changes to biotic interactions. These have been well reviewed elsewhere [1,4,7,38].

Trait plasticity is determined by a combination of factors including phylogenetic constraints, degree of niche specialisation, spatial scale of operation, behavioural flexibility, microhabitat adjustments, and physiological tolerance ranges (e.g., [39]). It must be considered, however, that while these changes are initially behaviourally and physiologically mediated within an individual, the degree of plasticity may have an additive genetic component. If plastic responses are favoured by selection via increasing survival and reproduction, these responses may become fixed in populations over time. Although species with an increased tolerance to thermal extremes might not necessarily have lower fitness under non-stressful conditions [40,41], there are potentially large costs associated with immediate plastic responses exhibited by species. For instance, acclimation that increases thermal resistance to high and low temperature extremes can have very large costs under non-stressful thermal environments [42].

It is likely that two prerequisites (caveats) must exist for adaptive responses and resilience to be successful in countering the rapid predicted change in global temperatures. These are biogeographic connectivity to allow organisms to reach suitable habitat/climate space/refugia and adequate time to allow adaptive changes. Lastly, any evolutionary shifts may be preceded by a reduction in population size [32] that puts the species at further risk of indirect effects of climate change, including effects of changing prey base, phenological mismatch, habitat type and extent, predators and disease pressure, and so on.

## Exposure

The exposure to climatic changes will depend on the degree of regional climatic change that occurs across the range of the organism or habitat in question and the degree to which local microhabitat buffering might reduce exposure (orange panels, Figure 1). A species might be buffered from the full magnitude of regional climate change by living in a thermally sheltered microhabitat such as boulder fields or under logs or use behavioural adjustments to regulate body temperature [43]. For example, possums reduce exposure to extreme temperatures by actively choosing to den in tree hollows that are 1.6 °C cooler during the day than other potential den locations [44]. Microhabitat buffering is at present a poorly recognised consideration in assessing vulnerability. Buffered microclimates associated with specific microhabitats may act as a mediating factor that confounds the straightforward interpolation from regional climate predicted by general circulation model to conditions experienced by the organism [45].

Our understanding of exposure to climate change is becoming increasingly refined by analytical techniques that allow conditions within microhabitats to be characterised (e.g., [46]) and by a combination of higher-resolution regional climate models and increasingly sophisticated spatial modelling of species distributions and demographics [47,48]. Increasingly, mechanistic models that incorporate physiological tolerances and energy constraints are being developed to make spatial predictions of how species will respond to changes in regional climate (e.g., [49–52]). However, mechanistic models can only be scaled up where general circulation model predictions are coupled to conditions directly experienced by organisms at finer spatial scales. Therefore, both a mechanistic understanding of factors that currently limit distribution [53] and knowledge of how organisms regulate exposure to extreme conditions at the microhabitat scale will be fundamental to understanding likely performance under future conditions. More robust predictions will emerge from a synergistic blend of these approaches.

The combination of exposure and sensitivity will determine the vulnerability of a species and thereby allow prediction of potential impacts on the species/habitat/process in question (pink/light red panels, Figure 1). Integrating and quantifying the complex parameters that contribute to relative vulnerability in a robust manner that takes into account all of the above discussed factors affecting relative vulnerability, predicted climatic change, and uncertainty will not be easy and represents a significant challenge.

## Feedbacks and Cascading Impacts

All realised impacts will have additional flow-on impacts via changes in assemblage composition, losses of genetic diversity, and changes to biotic and/or abiotic interactions with other species and ecosystem processes (Figure 1). These feedback impacts are potentially very significant in determining the impacts of climate change (blue panel, Figure 1). However, while these feedback effects and impact cascades will invariably alter the sensitivity, and perhaps even the exposure, of many species, the nature of such cascades and feedback effects is almost impossible to predict. Feedbacks and cascading impacts are likely to change community structure and composition, as well as species interactions such as competition, predator–prey relationships, parasitic infections, and decoupling of mutualisms [1,54]. Species-specific differences in sensitivity will undoubtedly result in the formation of novel assemblages [55] and changes to the functional roles of each species within the ecosystem. An increase in weedy and opportunistic species is also expected along with decoupling of phenological interactions [56] and changes in ecosystem processes such as primary productivity, decomposition, nutrient cycling, and fire regimes. Prediction of species vulnerability must be placed into a context of changing ecosystems and processes. However, the complexity and difficulty in predicting feedback impacts is considerable, and these feedback impacts will most likely need to be managed through a combination of monitoring and adaptive response with short- to medium-term objectives that are constantly re-evaluated. There are a number of ecosystem processes that can be evaluated immediately, and some progress has already been made in this regard (e.g., primary productivity and fire regimes [57,58]).

## Potential for Adaptive Management To Reduce Realised Impacts

The only truly effective method of minimising exposure is by the reduction of global greenhouse gas emissions (green panels, Figure 1). Without a significant reduction in the output of these gases, regional and species-specific management plans are doomed in the long term as climate change will continue. No matter what mitigation measures are put in place, lag effects will result in increasing impacts over the remainder of this century [6,59]. Therefore, regardless of future emission scenarios, understanding vulnerability will be a necessary part of planning for adaptation and resilience management. Assessing relative vulnerability can play a number of roles in this context. Firstly, research and management efforts can be directed and prioritised. Secondly, robust predictions of impacts that take into account all aspects of vulnerability can be used to estimate acceptable mitigation levels to inform government policy.

Locally, there may be some capacity to ameliorate impacts through targeted management actions that decrease exposure or increase resilience of the most vulnerable taxa or ecosystems, for example deployment of shade cloth shelters to lower nest temperatures [52]. Optimising the allocation of management effort requires a balance of the perceived threat/vulnerability, rate of change, consequences of inaction, social/political/scientific will, and available resources or management tools. Such management options could include, for example, maintenance of canopy cover and buffered microhabitats such as fallen debris through fire management, or the provision of thermally buffered shelters such as nest boxes for high-risk birds and mammals. In order to maximise the resilience of natural ecosystems, resource management must focus on maintaining healthy ecological processes and functions and minimise actions that may damage the inherent ability of the ecosystem to recover. This includes the full complement of standard conservation management practices such as minimising human impacts in potential refugial areas, minimising habitat loss, reducing fragmentation and maximising connectivity, and minimising the impacts of introduced animals, plants, and diseases. Additionally, we need to develop more refined tools for conservation planning that account for a changing climate.

Systematic conservation planning is an inherently spatial problem; however, climatic space is shifting and our traditional reserve systems are static. Given the current rate of climatic change, it will be impossible to maintain traditional approaches to conservation planning. Clearly, different components of an ecosystem may respond at different rates (e.g., structural shifts such as woody plant encroachment may take a long time, while fire regimes may change quite quickly). This introduces an added complication for policy makers and resource managers. If we wish to minimise the impacts of climate change on biodiversity, we need to develop dynamic approaches to conservation planning that integrate the different levels of vulnerability, rates, and spatial patterns of shifting biodiversity and the full spectrum of reserve and non-reserve preservation tactics. This will necessitate robust predictions of the future (what changes should we expect and how soon) because it is now essential that we move to a proactive approach to systematic conservation management rather than the more traditional reactive approach that is currently used within most management agencies.

## Concluding Statement

We have provided a conceptual framework that uses the best available information to clarify exactly how individual factors are expected to interact to yield a particular outcome for vulnerability under climate change. The next great challenge will be to apply the theoretical structure of the framework to derive quantitative estimates of vulnerability across a broad range of taxa. That is, it will be necessary to design a practical set of algorithms that describe interactions between traits/factors identified in the framework and assign sensible values (observed or inferred) to the input parameters to estimate vulnerability. We are encouraged by the fact that some quantitative links in the framework are already beginning to be forged [48,50,52]. The framework will ultimately enable comprehensive assessments of relative vulnerability (species, habitats, and processes). This understanding of the biological mechanisms underlying vulnerability will allow natural resource managers to determine the most efficient allocation of resources and researchers to identify important gaps in knowledge. With the requisite information, optimising the allocation of management effort will balance the perceived threat/vulnerability, rates of change, consequences of inaction, social/political/scientific will, and available resources/management tools [60].

## References

[pbio-0060325-b001] Hughes L (2000). Biological consequences of global warming: Is the signal already apparent. Trends Ecol Evol.

[pbio-0060325-b002] Walther G-R, Hughes L, Vitousek P, Stenseth NC (2005). Consensus on climate change. Trends Ecol Evol.

[pbio-0060325-b003] Root TL, MacMynowski DP, Mastrandrea MD, Schneider SH (2005). Human-modified temperatures induce species changes. Proc Natl Acad Sci U S A.

[pbio-0060325-b004] Parmesan C (2006). Ecological and evolutionary responses to recent climate change. Annu Rev Ecol Evol Syst.

[pbio-0060325-b005] Pounds AJ, Bustamante MR, Coloma LA, Consuegra JA, Fogden MPL (2006). Widespread amphibian extinctions from epidemic disease driven by global warming. Nature.

[pbio-0060325-b006] Parry ML, Canziani OF, Palutikof JP, van der Linden PJ, Hanson CE (2007). Climate change 2007: Impacts, adaptation and vulnerability. http://www.ipcc.ch/ipccreports/ar4-wg2.htm.

[pbio-0060325-b007] Parmesan C, Yohe G (2003). A globally coherent fingerprint of climate change impacts across natural systems. Nature.

[pbio-0060325-b008] Root TL, Price JT, Hall KR, Schneider SH, Rosenzweig C (2003). Fingerprints of global warming on wild animals and plants. Nature.

[pbio-0060325-b009] Rosenzweig C, Karoly D, Vicarelli M, Neofotis P, Wu Q (2008). Attributing physical and biological impacts to anthropogenic climate change. Nature.

[pbio-0060325-b010] Williams SE, Bolitho EE, Fox S (2003). Climate change in Australian tropical rainforests: An impending environmental catastrophe. Proc R Soc Lond B.

[pbio-0060325-b011] Thomas CD, Cameron A, Green RE, Bakkenes M, Beaumont LJ (2004). Extinction risk from climate change. Nature.

[pbio-0060325-b012] Root TL, Schneider SH (1995). Ecology and climate: Research strategies and implications. Science.

[pbio-0060325-b013] Root TL, Schneider SH (2006). Conservation and climate change: The challenges ahead. Conserv Biol.

[pbio-0060325-b014] Deutsch CA, Tewksbury JJ, Huey RB, Sheldon KS, Ghalambor CK (2008). Impacts of climate warming on terrestrial ectotherms across latitude. Proc Natl Acad Sci U S A.

[pbio-0060325-b015] McKinney ML (1997). Extinction vulnerability and selectivity: Combining ecological and paleontological views. Annu Rev Ecol Syst.

[pbio-0060325-b016] Bradshaw WE, Holzapfel CM (2006). Evolutionary response to rapid climate change. Science.

[pbio-0060325-b017] Skelly DK, Joseph LN, Possingham HP, Freidenburg LK, Farrugia TJ (2007). Evolutionary responses to climate change. Conserv Biol.

[pbio-0060325-b018] Smith B, Burton I, Klein RJT, Wandel J (2000). An anatomy of adaptation to climate change and variability. Clim Change.

[pbio-0060325-b019] Turner BL, Kasperson RE, Matson PA, McCarthy JJ, Corell RW (2003). A framework for vulnerability analysis in sustainability science. Proc Natl Acad Sci U S A.

[pbio-0060325-b020] Schröter D, Brussaard L, De Deyn G, Poveda K, Brown VK (2004). Trophic interactions in a changing world: Modelling aboveground-belowground interactions. Basic Appl Ecol.

[pbio-0060325-b021] Chown SL, Gaston KJ (2008). Macrophysiology in a changing world. Proc R Soc Lond B.

[pbio-0060325-b022] Addo-Bediako A, Chown SL, Gaston KJ (2000). Thermal tolerance, climatic variability and latitude. Proc R Soc Lond B.

[pbio-0060325-b023] Calosi P, Bilton DT, Spicer JI (2008). Thermal tolerance, acclimatory capacity and vulnerability to global climate change. Biol Lett.

[pbio-0060325-b024] Stillman JH (2003). Acclimation capacity underlies susceptibility to climate change. Science.

[pbio-0060325-b025] Chown SL, Addo-Bediako A, Gaston KJ (2002). Physiological variation in insects: Large-scale patterns and their implications. Comp Biochem Physiol.

[pbio-0060325-b026] Blomberg SP, Garland T, Ives AR (2003). Testing for phylogenetic signal in comparative data: Behavioural traits are more liable. Evolution.

[pbio-0060325-b027] Brattstrom BH (1970). Thermal acclimation in Australian amphibians. Comp Biochem Physiol.

[pbio-0060325-b028] Blows MW, Hoffmann AA (2005). A reassessment of the genetic limits to evolutionary change. Ecology.

[pbio-0060325-b029] Willi Y, Van Buskirk J, Hoffmann AA (2006). Limits to the adaptive potential of small populations. Annu Rev Ecol Evol Syst.

[pbio-0060325-b030] Jamieson IG, Wallis GP, Briskie JV (2006). Inbreeding and endangered species management: Is New Zealand out of step with the rest of the world. Conserv Biol.

[pbio-0060325-b031] Fjerdingstad E, Schtickzelle N, Manhes P, Gutierrez A, Clobert J (2007). Evolution of dispersal and life history strategies—Tetrahymena ciliates. BMC Evol Biol.

[pbio-0060325-b032] Gomulkiewicz R, Holt RD (1995). When does evolution by natural selection prevent extinction. Evolution.

[pbio-0060325-b033] Berger R, Lynch M (1995). Evolution and extinction in a changing environment—A quantitative-genetic analysis. Evolution.

[pbio-0060325-b034] Kellermann VM, Van Heerwaarden B, Hoffmann AA, Sgro CM (2006). Very low additive genetic variance and evolutionary potential in multiple populations of two rainforest Drosophila species. Evolution.

[pbio-0060325-b035] Etterson JR, Shaw RG (2001). Constraint to adaptive evolution in response to global warming. Science.

[pbio-0060325-b036] Hellmann JJ, Pineda-Krch M (2007). Constraints and reinforcement on adaptation under climate change: Selection of genetically correlated traits. Biol Conserv.

[pbio-0060325-b037] Wilson AJ, Pemberton JM, Pilkington JG, Coltman DW, Mifsud DV (2006). Environmental coupling of selection and heritability limits evolution. PLoS Biol.

[pbio-0060325-b038] Walther G-R, Post E, Convey P, Menzel A, Parmesan C (2002). Ecological responses to recent climate change. Nature.

[pbio-0060325-b039] Nylin S, Gotthard K (1998). Plasticity in life history traits. Annu Rev Entomol.

[pbio-0060325-b040] Angilletta MJ, Wilson RS, Navas CA, James RS (2003). Tradeoffs and the evolution of thermal reaction norms. Trends Ecol Evol.

[pbio-0060325-b041] Huey RB, Kingsolver JG (1993). Evolution of resistance to high temperature in ectotherms. Am Nat.

[pbio-0060325-b042] Kristensen TN, Hoffmann AA, Overgaard J, Sorensen JG, Hallas R (2008). Costs and benefits of cold acclimation in field-released Drosophila. Proc Natl Acad Sci U S A.

[pbio-0060325-b043] Stevenson RD (1985). The relative importance of behavioral and physiological adjustments controlling body temperature in terrestrial ectotherms. Am Nat.

[pbio-0060325-b044] Isaac JL, De Gabriel JL, Goodman BA (2008). Microclimate of daytime den sites in a tropical possum: Implications for the conservation of tropical arboreal marsupials. Anim Conserv.

[pbio-0060325-b045] Kennedy AD (1997). Bridging the gap between general circulation model (GCM) output and biological microenvironments. Int J Biometeorol.

[pbio-0060325-b046] Helmuth B (2002). How do we measure the environment? Linking intertidal thermal physiology and ecology through biophysics. Integ Comp Biol.

[pbio-0060325-b047] Elith J, Graham CH, Anderson RP, Dudik M, Ferrier S (2006). Novel methods improve prediction of species' distributions from occurrence data. Ecography.

[pbio-0060325-b048] Keith DA, Akçakaya HR, Thuiller W, Midgley GF, Pearson RG (2008). Predicting extinction risks under climate change: Coupling stochastic population models with dynamic bioclimatic habitat models. Biol Lett.

[pbio-0060325-b049] Hijmans RJ, Graham CH (2006). The ability of climate envelope models to predict the effect of climate change on species distributions. Global Change Biol.

[pbio-0060325-b050] Kearney M (2006). Habitat, environment and niche: What are we modelling. Oikos.

[pbio-0060325-b051] Logan JA, Régnière J, Gray DR, Munson AS (2007). Risk assessment in the face of a changing environment: Gypsy moth and climate change in Utah. Ecol Appl.

[pbio-0060325-b052] Mitchell NJ, Kearney MR, Nelson NJ, Porter WP (2008). Predicting the fate of a living fossil: How will global warming affect sex determination and hatching phenology in tuatara. Proc R Soc Lond B.

[pbio-0060325-b053] Kearney M, Porter WP (2004). Mapping the fundemental niche: Physiology, climate, and the distribution of a nocturnal lizard. Ecology.

[pbio-0060325-b054] Sutherst RW, Maywald GF, Bourne AS (2007). Including species interactions in risk assessments for global change. Global Change Biol.

[pbio-0060325-b055] Davis MB, Shaw RG (2001). Range shifts and adaptive responses to Quaternary climate change. Science.

[pbio-0060325-b056] Visser ME, Both C (2005). Shifts in phenology due to global climate change: The need for a yardstick. Proc R Soc Lond B.

[pbio-0060325-b057] Clark DA, Piper SC, Keeling CD, Clark DB (2003). Tropical rain forest tree growth and atmospheric carbon dynamics linked to interannual temperature variation during 1984–2000. Proc Natl Acad Sci U S A.

[pbio-0060325-b058] Swetnam TW (1993). Fire history and climate change in giant sequoia groves. Science.

[pbio-0060325-b059] Graham NAJ, Wilson SK, Jennings S, Polunin NVC, Robinson JAN (2007). Lag effects in the impacts of mass coral bleaching on coral reef fish, fisheries, and ecosystems. Conserv Biol.

[pbio-0060325-b060] Marsh H, Dennis A, Hines H, Kutt A, McDonald K (2007). Optimizing allocation of management resources for wildlife. Conserv Biol.

